# Functional Foods and Bioactive Compounds: A Review of Its Possible Role on Weight Management and Obesity’s Metabolic Consequences

**DOI:** 10.3390/medicines6030094

**Published:** 2019-09-09

**Authors:** Melina Konstantinidi, Antonios E. Koutelidakis

**Affiliations:** Department of Food Science and Nutrition, University of the Aegean, Myrina, 81400 Lemnos, Greece

**Keywords:** functional foods, bioactive compounds, weight management, obesity, metabolic consequences

## Abstract

**Background:** Weight management and obesity prevention is a basic aim of health organizations in order to decrease the prevalence of various metabolic disorders. The aim of the present review article was the evaluation of the possible role of functional foods and their bioactive compounds as alternative way to promote weight management and prevent obesity and its metabolic consequences. **Methods:** Approximately 100 articles were selected from Scopus, PubMed, Google Scholar, and Science Direct, by using relative key words, and based mainly on recent animal, clinical or epidemiological studies. **Results:** The literature review highlighted the possible effect of specific functional foods such as coffee, green tea, berries, nuts, olive oil, pomegranate, avocado, and ginger. Specific bioactive compounds of those foods—such as caffeine, catechins, gallic acid, anthocyanins, ascorbic acid, polyphenols, oleuropein, capsaicin, and quercetin—may contribute to weight management, obesity prevention, and obesity’s metabolic consequences. The possible mechanisms include effect on satiety, lipid absorption, fatty acids beta oxidation, stimulation of thermogenesis, etc. **Conclusions:** Functional foods, as part of a balanced diet, could be useful in the direction of weight management and decrease of obesity’s’ metabolic consequences. However, the scientific evidence is unclear and in most cases controversial and more clinical and epidemiological studies are needed in order to further investigate the mechanisms of their possible effect.

## 1. Introduction

Obesity defined by the World Health Organization (WHO) as a condition characterized by inordinate accumulation of body fat and as a public health problem that lead to serious social, physical, and psychological problems. The body mass index (BMI) of 30 kg/m^2^ is the cut-off point for obesity, which have associated with increased risk for chronic diseases, morbidity, and mortality [1]. According to European studies, a growing health problem includes child obesity, especially in countries around the Mediterranean Sea that have shown very high rates of overweight and obesity. Surveys in Greece have noted that child obesity levels are the highest in Europe [2]. Although food portion control is very important issue for weight management, the exhortation of people to eat less of all foods may not lead to desirably results, given the fact that the high-energy-dense foods disproportionately increase energy intake in comparison with those with lower content in energy density. A more adequate strategy might be the encouragement of people increasing the consumption of low-energy-dense foods and limiting the consumption of high-energy-dense foods, in parallel with following a balanced diet, such as the Mediterranean diet, in combination with increase of physical activity [3].

Functional foods (FFs) were first defined in the 1980s, when the Ministry of Health and Welfare of Japan established a regulatory system for foods that possess possible health benefits. A food can be considered as ‘functional’ if it possesses constructive effects on target functions into the human organism, beyond nutritional effects, with aim of health promotion and wellbeing and/or the reduction of chronic diseases. Functional foods are attaining eminence globally and are part of the daily diet of consumers. By 2020, functional foods’ and beverages’ global market has been predicted to be worth $192 billion [4]. Functional foods are often indicated as ‘natural health products’ or ‘healthy foods’. Although there is not a unique definition worldwide, foods can only be considered functional if, along with basic nutritional impact, they exhibit possible beneficial effects on specific functions in the human organism with the result of improving the physical condition and/or decreasing the risk of chronic disease appearance [5,6]. However, nutritional and health claims about the possible effect of functional foods on disease prevention has been established only in specific situations, when sufficient scientific data ensure their properties. Furthermore, the scientific research concludes that functional foods may have beneficial effects in human health only if they are part of a balanced diet, such as the Mediterranean diet [7,8,9].

Bioactive compounds are present in small amounts into the food items and their effect on human health is being continuously investigated. Epidemiological data support that high intake of natural functional foods, such as specific fruits and vegetable, which are rich in bioactive compounds, is associated with decreased risk of chronic diseases, such as cardiovascular diseases, cancer, metabolic syndrome, diabetes type II, and obesity [10]. Natural bioactive compounds—such as resveratrol, epigallocatechin, curcumin, oleuropein, sulforaphane, quercetin, ellagic acid, anthocyanins, b-glucans, and other biomolecules—have been studied as factors with possible direct or indirect effect on specific molecular pathways, associated with the pathophysiology of cardiovascular diseases, diabetes, metabolic syndrome, and cancer. However, more clinical and epidemiological studies are essential in order to ensure their possible effect [7,8,9], given the fact that the conducted studies have different experimental designs and their result are, in many cases, controversial. Resveratrol (3,5,4-trihydroxystilbene) (RSV) has been detected mainly in grapes, red wine, and pomegranates and it is the main stilbene present in human diet. Epigallocatechin-3-gallate (EGCG) is the major polyphenol from green tea with high antioxidant and scavenging properties. It is the ester of gallic acid with epigallocatechin and characterized by two triphenolic groups. Curcumin (CUR) exhibit possible anti-inflammatory, antioxidant and chemo-preventive actions and it is a polyphenol from *Curcuma longa* Linnaeus (Zingiberaceae). Quercetin (QR) is a flavonol found in various foods, such as berries and tea; its antioxidant activity is the result of three important groups: the 4-oxo group in combination with the 2,3-alkene, 3- and 5-hydroxyl groups, and the hydroxyl groups of B ring. Ellagic acid (EA) applies high antioxidant and radical scavenging activity and it is a polyphenol derived from ellagitannins mostly found in nuts and berries. Anthocyanins (ACs) are water-soluble pigments with purple, red, and blue colors and present in fruits, especially in pomegranate, berries, and red grapes. Anthocyanins have studied as bio-functional molecules with possible high anti-inflammatory, antioxidant, and chemoprotective effects [11].

Obesity is a metabolic disease, which has become a problem of epidemic spread at European and global level and is related with other chronic degenerative or inflammatory diseases and metabolic consequences [7]. Increased food consumption, decreased physical activity, positive energy balance, and food addiction are parameters that may lead to increased obesity levels globally. The often consumption of foods that are of high content in sugar and fat, in combination with strong cravings or compulsions, is characterized as food addiction [12,13]. The strategies for weight control management and obesity prevention or treatment include increase of energy expenditure and following of a balanced diet in order to achieve negative energy balance. In recent years, it has been observed that an increase in efforts has been made for finding and applying alternative ways to prevent obesity, in order to decrease the impact and the cost of its metabolic consequences. The scientific community, based mainly on clinical and epidemiological studies, highlights the importance and the possible health effects of certain foods, so-called functional foods. The incorporation into a diet of several natural or processed foods with possible health effects, such as functional foods, may contribute to the weight management, if they are combined with the following of a balanced diet [7,8]. In recent study, with 300 healthy volunteers, the authors noticed a decreased BMI in those who often consumed gojiberry, cranberry, and pomegranate in contrast to those who had never consumed these functional foods [7]. In addition, scientific data support that the consumption of specific functional foods and bioactive compounds, such as b-glucane, glucomanane, foods with reduced fat and sugar content, etc., may contribute to weight management and metabolic consequences in obese people and could be part of a balanced diet for obesity management or treatment. However, the scientific data underline that these specific functional foods and bioactive compounds cannot contribute to weight control if consumed unilaterally and in large quantities.

The aim of the present review article was to conduct a critical literature survey in order to present, highlight, and discuss recent studies about the possible role and effect of specific bioactive compounds and functional foods on weight management, and obesity’s metabolic consequences, including cardiovascular diseases, hypertension, metabolic syndrome, and diabetes mellitus. For that scope, approximately 100 articles were selected from Scopus, PubMed, Google Scholar, and Science Direct, by using relative key words, and based mainly on recent animal, clinical, or epidemiological studies. The 100 articles selected from a larger number of articles, with the use of combinations of specific key words—such as ‘obesity’, ‘metabolic disorders’, and ‘functional foods’—and the names of bioactive compounds and functional foods that their role on weight control have studied the last years. The selection performed by specific criteria and specifically: a) recent articles published mainly the last decade; b) articles which present animal experiments, human clinical trials, and epidemiological studies; c) selection of functional foods that are studied as possible factors for weight management in an important number of recent studies.

## 2. Recent Data about The Possible Effect of Specific Functional Foods and Their Bioactive Compounds on Weight Control and Obesity’s Metabolic Consequences

Animal experiments as well as clinical studies have been performed in order to investigate the possible effect of functional foods and bioactive compounds on weight control and the metabolic consequences of obesity. The studies present different conclusions due to different experimental designs and target groups and the results, in some cases, are unclear and controversial. Most studies present some indications, but no clear evidence. The results from animal studies lead to different and no clear results. In a recent study, the consumption of 68.3 mg/kg decaffeinated green coffee beans from 40 male rats for 11 weeks resulted in body weight gain and increased cardiovascular diseases [14]. However, in another study the consumption of 100 mg capsules or powder of green tea from 10 male rats for 30 days led to reduced body weight gain [15]. In a recent study has been observed that the consumption of 20–60 mg/day oleuropein powder from 8 male rats for 4 weeks led to increased blood pressure and glucose [16]. Other study concluded that the consumption of 2 g avocado from 35 male rats for 7 weeks led to increased cholesterol but reduced body weight, blood glucose, BMI, and serum lipids [17]. Finally, the consumption of 500 mg/kg ginger from rats for 30 days led to increased liver weight but decreased plasma glucose levels [18].

Table 1 present selected animal studies about the possible effect of functional foods in weight control, obesity, and its metabolic consequences (glucose and lipids levels). The animal studies could present a first indication about the possible role of some functional foods on weight control; however, human studies are essential for safer investigation. The animal experiments in obesity models were performed with various experimental designs, doses of bioactive compounds, and metabolic diseases; so the results could not be reliable for general conclusions.

### 2.1. Coffee and Green Coffee

Coffee is one of the major non-alcoholic beverages and most widely consumed pharmacologically active beverages worldwide. Green beans of coffee are essentially non-roasted coffee beans and green coffee grounds can be viewed as one of the most important types of grain traded worldwide [23,24]. Coffee cultivation began in Arabic from Ethiopia centuries ago; it continued to flourish in countries such as Africa, Latin America, and Asia. Coffee is often composed by a variety of crops of roasted beans and consists of two species, *Coffea canephora* and *Coffea Arabica*, which can be found in organic and conventional types [25]. Nowadays, coffee is the second most traded commodity worldwide and has emerged as a cultural phenomenon with the consumption of hundreds of billions of cups every year [26].

According to the Dietary Guidelines Advisory 2015 it is noted that people should consume up to about 400 mg dose of coffee in either capsule or powdered form—i.e., about 2–3 coffee cups per day. In a recent study the authors observed in healthy adults that moderate coffee consumption (3–5 cups/day or up to 400 mg/day caffeine) was not associated with increased risk of major chronic diseases prevalence, such as cardiovascular disease, premature death and cancer [27]. However, the scientific data support that, above this level, consumption exhibits negative consequences.

#### 2.1.1. Bioactive Compounds of Coffee and Green Coffee

One of the most important bioactive components of coffee is the methyl-xanthine alkaloid caffeine, which consist the 1.2–2.2% of coffee. Caffeine is a dietary compound which appears stimulant action, is not a nutrient and occurs in plants like coffee beans. Chlorogenic acids are other important bioactive compounds, found especially in green coffee, in *Coffea arabica* and *Coffea canephora* seeds and include caffeoylquinic acid, dicaffeoylquinic acid, feruloylquinic acid, p-coumaroylquinic acid, and caffeolyl-feruloylquinic acid. During the roasting process, roast coffee is rich in a variety of compounds which provide aroma, flavor, and color due to the chemical reactions [15,27]. Roasting coffee contains plethora of bioactive compounds and its chemical composition is the following: 2.7–3.1% chlorogenic acid, 1.2–2.4% caffeine, 23% melanoidins, 11–17% lipids, 38–42% carbohydrates, 10% proteins, and 2.4–2.5% aliphatic acids. The roasting process increases the soluble dietary fiber from 39.4 mg per 100 g in green beans to 64.9 mg per 100 g in roasted beans. The chemical composition of green coffee beans before roasting process is the following: 6.5–10% chlorogenic acid, 1.2–2.2% caffeine, 10–16% lipids (mainly the diterpenes cafestol and Kahweol), 0.7–1.0% trigonelline, 45–52% carbohydrates, 11% proteins, and 4.2–4.4% minerals [15].

#### 2.1.2. Possible Effect of Coffee and Green Coffee on Human Health

Scientific data conclude that moderate coffee consumption is associated with decreased risk of diabetes mellitus type II and cardiovascular disease in adults. Moreover, some evidence indicates possible association between caffeine intake and reduced risk of Parkinson’s disease [27]. The frequent consumption of a green or roasted coffee may be beneficial to healthy and hypercholesterolemic subjects in order to avoid metabolic syndrome (MetS), as it may exhibit positive effects on blood pressure, serum glucose and triglyceride levels, being especially interesting for subjects with hypercholesterolemia, as coffee improved the lipid profile [28]. In another study 68.3 mg/kg dose of coffee was administrated to 40 male rats with metabolic syndrome, cardiovascular and hepatic structure for 8–9 weeks and concluded increased cardiovascular diseases [14]. In a randomized control blinded trial, the consumption of 500 mg dose of caffeine in capsules or powder form to 137 patients with arrhythmic episodes for 2 months and resulted no arrhythmic episodes [29]. However, other studies have recently underlined the possible negative effects of the increased consumption, such as cardiac arrhythmia, etc.

#### 2.1.3. Possible Effect of Coffee and Green Coffee on Weight Control

In Table 2 recent human interventional clinical studies about the possible effect of coffee on body weight control and diseases prevention are summarized. Although, several scientific studies support that green coffee consumption may contribute to weight management the scientific data remain unclear. In a recent study green coffee bean extract significantly decreased body weight and fat mass. The authors concluded that in addition to the antioxidant activity of coffee, coffee consumption may possible lead to significantly declined body weight and body fat [26]. In a randomized controlled trial the consumption of 3–5 cups/day of coffee for 3 weeks in 25 males and 95 females with obesity led to contribution to the weight control and increased number of *Bifidobacterium spp* [15,27]. In onother study the authors used coffee silverskin from Arabic and Robusta varieties (*Coffea Arabica* and *Coffea canephora,* respectively) as raw materials. The main bioactive component that used for comparative analysis was tablets of decaffeinated green coffee extract and CGA (5-caffeoyl quinic acid). About beverages, there were bags containing samples and were prepared using 10–20 μm filter papers and infused in boiling water for different time points. Brewing conditions were optimized by studying influence of: (a) raw material; (b) extraction time, 5 and 10 min; (c) concentration of raw material (2.5, 5, and 10 mg/mL); and (d) coffee variety. They observed in humans that coffee consumption prevented fat accumulation and excess weight [30]. In several studies, the green coffee bean extract showed stronger effect on body weight reduction and on decrease of epididymal and perirenal fat weight than roasted coffee and caffeine, possibly due to its high content on chlorogenic acids. Coffee may also influence gut microbiota in animals and humans with obesity. Although the possible anti-obesity mechanism of coffee still needs further investigation, it might have prospective as a new strategy for weight management [15]. We can summarize that coffee is one of the beverages that some indications exist about its role on weight management. Although consumption of coffee and green coffee led to weight control in several studies, especially by reduction in BMI, further studies are needed in order to fully investigate the possible effects on weight management and human health.

### 2.2. Tea and Green Tea

Many studies support that polyphenols in tea may contribute to reducing cardiovascular disease and type II diabetes mellitus, lowering risk of obesity, lowering cholesterol, and low-density lipoprotein (LDL), as well as having anti-atherosclerotic properties and it may reduce various forms of cancer. Laboratory and animal studies showed that green tea, and in particular EGCG, seems to help control weight by reduction of adipocyte proliferation, impeding the absorption of fat and reduction of blood triglycerides, cholesterol, glucose, and insulin [36,37,38,39,40]. According to the literature review, green tea is the functional beverage with the most studies conducted and with some clear evidence about its possible effect on weight control; however, more studies are needed for safer results.

#### 2.2.1. Bioactive Compounds of Green Tea

Green tea constitutes a beverage rich in polyphenolic compounds, especially epigallocatechin gallate (EGCG), catechin (C), galactatechin (GC), epigallocatechin (EGC), epicatechin (EC), and galactoatechin gallate (GCG). Tea bioactivity is based mainly on the antioxidant activity of tea polyphenols and is affected from various nutritional and physiological parameters [41]. Although the majority of clinical studies are not designed for safety evaluation of dietary bioactive compounds, safety results of great value are reported in clinical efficacy studies and are used to establish the efficacy levels of bioactive compounds on target populations [36]. The antioxidant mechanism of tea polyphenols has been determined by in vitro and in vivo analyses. The fermentation process affects tea bioacative components and thus changes its antioxidant activity. In a recent study the authors used Taiwanese tea and especially four types—which include green (non-fermented), oolong (semi-fermented), black (fully fermented), and Pu-erh (post-fermented) tea—at different degrees of fermentation and determined the polyphenol levels by high performance liquid chromatography (HPLC). The study observed a clear correlation between gallic acid content and the degree of fermentation; the lowest gallic acid concentration was 1.67mg/g in Pu-erh tea, while the highest was 21.98 in green tea [38].

#### 2.2.2. Possible Effects of Tea on Weight Control: Possible Mechanisms

Animal and human studies have shown that tea catechins may help on weight control but the results are controversial. Treatment of rats with EGCG indicated reduction on food intake, while EGCG has proposed as a factor with possible contribution on weight loss. Green tea catechins (GTC) attenuated the increase on body and liver weights and improved serum and liver triglyceride levels. Rats fed a chow diet had more weight reduction than rats fed a high fat diet, while GTC supplementation significantly decreased body weight [37]. Although some animal studies showed possible effect on weight control and especially led to reduce weight control, human studies have not fully ensured these results. More epidemiological studies with large sample may supplement the existing indications about the role of green tea on weight management. However, the scientific data about the effect of tea on metabolic biomarkers, such as cardiovascular and diabetic indexes are clearer and show possible positive effects.

Two possible mechanisms of green tea polyphenols effect on weight management are: (i) tea components may decrease lipids’ and proteins’ absorption into the intestine, consistently reducing calorie intake; and (ii) tea polyphenols activate AMP-activated protein kinase which is bioavailable in liver, skeletal muscle, and adipose tissue. The importance of these two mechanisms is depended on the tea type and the consumed diet by individuals [42]. Possible beneficial effects on body-weight management have been observed from mixing green tea catechins with caffeine; studies showed maintenance or reduction of body weight, which possibly caused by increase on energy expenditure, thermogenesis and fat-oxidation [43,44,45,46]. In other studies the consumption of 100 mg tea catechins in capsules or powder from 10 male rats with obesity for 30 days led to the reduction of body weight gain [14,37]. In a randomized control blinded trial, was observed that the consumption of 704 mg tea catechins per day from 40 males and 37 females with liver problem for 14 days did not have an adverse effect on liver [47]. A randomized controlled, blinded trial concluded that the consumption of 491 mg tea catechins per day in capsule form by 115 obese women for 12 weeks led to significant weight reduction [48]. In another study the authors administrated 90 mg epigallocachin-3 gallate in capsules form to 8 young men with thermogenesis and concluded that tea catechins stimulated thermogenesis [43].

Table 3 presents several human intervention clinical studies about the possible effects of green tea on weight management and disease prevention. Most of the studies used randomized and controlled design with consumption of about 3–5 cups tea per day for several weeks and showed a small reduction on weight or BMI and improvements on metabolic biomarkers.

### 2.3. Berries

#### 2.3.1. Bioactive Compounds of Berries

Berries are rich in phenolic compounds, such as anthocyanins, flavonols, flavanols, proanthocyanidins, ellagitannins, and phenolic acids, with anthocyanins being the stronger pigments, responsible for the red, blue, or purple color in berries, which act as powerful antioxidants. One kind of berries, aronia, contains polyphenols with anti-inflammatory activities. Scientific evidence support that aronia berry consumption may modulates intestinal immune function and T-cells [20,55].

Berries—such as blackberry, blueberry, strawberry, etc.—are one of the best dietary sources of bioactive components, which may exhibit a synergistic and cumulative effect on human health promotion and possible prevention of chronic diseases such as obesity, hypertension, type II diabetes mellitus, dyslipidemia, and nonalcoholic fatty liver disease. Berry consumption can be considered as an effective alternative way of dietary pattern modifications focused on decreasing the development of metabolic syndrome. Clinical studies have shown that the long-term consumption of berries in patients with metabolic syndrome may provide beneficial results to their health. Moreover, berry consumption may protect from cardiovascular metabolic consequences [56,57].

#### 2.3.2. Possible Effects of Berries on Weight Management and Obesity’s Metabolic Consequences

It has been observed that the administration of aronia berry in mice raised the colonic IL-10 secretion, while 4.5% of aronia berry did not constrain ex vivo stimulated cytokine production by colon and spleen tissue. Unstimulated colon tissue ex vivo excretion of IL-10 was raised by 100% in the aronia fed mice, in comparison to the control group (*p* = 0.0348). IL-17 and IL-6 were not importantly different in the aronia-fed mice (*p* = 0.0535 and *p* = 0.0850, respectively) [36]. According to animal studies and controlled clinical trials, a possible anti-obesity effect of berries was observed via a decrease in body fat and waist circumference. In several studies, berries suppressed increased liver and epididymal fat weight, fasting blood glucose, triglycerides, and LDL-C levels [56,57]. The administration of 500 mg dose of berries’ anthocyanins in powder form from 80 rats with chronic diseases for 7 weeks led to the reduction of metabolic chronic diseases [19]. In a randomized controlled blinded trialthe consumption of 100–140 mg/day of berries in capsules form from 63 participants with diabetes mellitus for 12 weeks increased the glycemic control [58]. In a randomized controlled trial the consumption of 163 g berries per day from 61 women for 20 weeks, increased fasting plasma HDL, and decreased systolic and diastolic blood pressure [59]. The consumption for 5 weeks of 414.2 mg/day berries’ anthocyanins from 40 healthy older adults with type II diabetes mellitus, improved type II diabetes consequences, and cardiovascular diseases’ biomarkers [57]. In another study, the complementation of a high-fat diet with berries prevented weight gain and declined insulin resistance [60]. The consumption of black berries (BB) led to reduced average 24 h respiratory quotient (RQ) (0.810 vs. 0.817, BB vs. gelatin, GEL, *p* = 0.040), increased fat oxidation and improved insulin resistance in overweight or obese males fed a high fat diet. RQ reduction correlated with 7% raise in fat oxidation (141 g/day for BB vs. 132 g/day for GEL, *p* = 0.0420); in the evening the fat oxidation increased to 14% (28 g for BB vs. 25 g for GEL, *p* = 0.0069), during the morning increased to 11% (21 vs. 19 g, BB vs. GEL, *p* = 0.0129), while during exercise raised to 12% (9 vs. 8 g, BB vs. GEL, *p* = 0.0044). A randomized crossover study with consumption of 600 g blackberries daily for one week, concluded that blackberry consumption significantly increased fat oxidation and decreased insulin resistance in overweight or obese men followed a high-fat diet in comparison with an energy matched control diet [61].

As a conclusion, we can underline that although many studies have shown the possible effect of berries on metabolic biomarkers, the studies about their effect on weight management are limited; more clinical and epidemiological studies are needed in a large number of individuals for a long time so as to draw more clear conclusions about the effect of berries on weight control.

### 2.4. Pomegranate

Pomegranate (*Punica granatum L.*) is rich in tannins, especially ellagitannins, as well as flavonoids such as catechins, epicatechin, quercetin, anthocyanins, and procyanidins, all bioactive compounds that may contribute to possible health benefits [62,63]. Pomegranate belongs to Punicaceae family and is a fruit with strong antioxidant activity, involving bioactive phenolic components with possible health benefits. Pomegranate peel accounts for 30–40% of the fruit and constitutes a juice byproduct, which is considered an unused material [64]. Pomegranate decreases fasting serum glucose levels as it contains anthocyanins and tannins such as punicalagin, ellagic, and gallic acids that might possess anti-diabetic actions. Pomegranate may affect type II diabetic pathophysiology by reduction of oxidative stress and lipid peroxidation [65]. Both pomegranate seeds and juice may contribute to the prevention of the most important chronic diseases, including cancer, cardiovascular disease, diabetes mellitus, and obesity [66].

Anthocyanins are considered of high importance among pomegranates’ bioactive phenolic compounds. Hydrolysable tannins, especially ellagitannins and gallotannins, form the most prevalent compounds in pomegranates, so they are present in all the parts of the plant. Pomegranates’ bioactivity is possibly the result of these compounds; the main pomegranate ellagitannins are punicalagins and granatins [63,64]. Moreover, pomegranates’ peels are used in fruit processing industry as new sources of bioactive compounds with possible utilize in food industry for novel functional foods production [64]. The consumption of 50 mg of powder pomegranate from 6 dogs led to positive impact on gut health [22]. The consumption of pomegranate leaf extract and 10–20 mg of ascorbic acid from pomegranate in capsules or powder form from a large sample led to the treatment of obesity and type II diabetes and decreased body weight [67]. The literature review showed that the studies about the possible role of pomegranate on weight loss are limited, while several studies have been performed about its role on obesity’s metabolic consequences.

Table 4 presents several human intervention studies about the possible effects of berries and pomegranate on body weight control, obesity, and its metabolic consequences.

### 2.5. Nuts and Seeds

#### 2.5.1. Bioactive Compounds of Nuts

Nuts and seeds are considered basic components of a healthy diet, such as the Mediterranean diet. According to some epidemiologic studies, adding nuts to a diet does not cause weight gain. Also, nuts intake reduced the risk of cardiovascular disease (CHD), reduced type II diabetes mellitus, and influenced risk of hypertension and risk of heart failure. With the exception of chestnuts, lipids are the main ingredients in nuts and seeds, followed by protein and carbohydrates; thus, nuts and seeds appear a high energy content [69,70]. Nuts contain increased amounts of nutrients including fiber, monounsaturated fatty acids (MUFA), polyunsaturated fatty acids (PUFA), vitamin E, folate, magnesium, potassium, and calcium and in parallel have decreased levels of saturated fatty acids (SFA). Walnuts, particularly, are an excellent source of a-linolenic acid, which is an important omega-3 fatty acid that decreases cardiovascular disease incidence and possibly the risk of cancer [71]. The main polyphenol in walnuts is pedunculagin, an ellagitannin, which is hydrolyzed after consumption, in order to release ellagic acid, which is converted by gut microflora to urolithin A and other substances, as urolithins B, C, and D. Ellagitannins (ETs) retain antioxidant and anti-inflammatory bioactivity and may contribute to the attenuation of the onset and progression of cancer, cardiovascular, and neurodegenerative diseases [72].

Nuts are made up of 50–75% fat, yielding an average of 5–7 kcal/g, of which the least is saturated, while most are monounsaturated and polyunsaturated, which places among high-nutrition foods. For instance, peanuts are rich in monounsaturated fat, with more than 80% oleic acid, while nuts are characterized by their high content of α-linolenic acid, a short chain omega-3 polyunsaturated acid. Nuts are also a rich source of protein (about 25% of their total energy), and for the peanuts there is an extremely high L-arginine content. Only 15% of their total energy comes from carbohydrates, while they are a rich source of fiber, ranging from 4 to 11% of total weight [73]. Therefore, nuts are foods with high energy content.

#### 2.5.2. Possible Effects of Nuts on Weight Control: Possible Mechanisms

In developed countries, there is an increase in overweight and obese people, especially adults, due to the increased energy consumption and unbalanced nutrition. In a recent studythe participants maintained levels of physical activity to ensure the stability of the weight. Also, the participants had the intention of replacing the fat or protein foods in their diet and consuming walnuts during the day as a snack. The study resulted reduction in systolic blood pressure, while no change on baseline anthropometric characteristics was observed [74]. In another study, a healthy diet containing one daily serving of nuts for 12 weeks had little effect on lipid profile of metabolic syndrome (MetS) patients but led to improvement on insulin sensitivity and a marginal anti-inflammatory effect, in combination with moderate weight loss. Therefore, regular nut consumption could help patients with MetS, although changes in cholesterol metabolism make them resistant to cholesterol lowering [75]

The consumption of 85.2 g of walnuts from 40 male rats with increased blood glucose level for 2 weeks reduced the blood glucose levels [21]. In a randomized control trial for 12 weeks the consumption of 30 g/day of cashew nuts from 300 adults with type II diabetes, increased HDL cholesterol, body weight, BMI, waist circumference, and decreased systolic blood pressure [76]. In other randomized control trial the consumption of 50 g of walnuts from 63 patients with prostate cancer or prostate hyperplasia improved prostate biomarkers [72]. The consumption of 30g of nuts from 50 patients with insulin resistance for 12 weeks led to decreased insulin resistance [75]. The consumption of 56 g of walnuts from 46 overweight, obese adults (28 women and 18 men) for 4 weeks concluded improvement of endothelial function in the overweight participants, but the body weight was unchanged [77]. In a randomized control study the consumption of 43 g of almonds from 137 participants with postprandial glycemia concluded that hunger suppressed [78]. All the above studies concluded that nuts consumption improved metabolic biomarkers and could be an ideal source of fat, by replacing saturated fatty acids.

Several studies support that because nuts are energy dense, they should be replaced from other foods into the diet in order to control calories and attenuate an excess energy intake and weight gain [74]. The high energy density of nuts may lead to the conclusion that their consumption promotes weight gain. However, the US Department of Agriculture’s Continuing Survey of Food Intakes concluded that BMI from 12,000 participants was lower among nuts consumers (23.8 ± 0.1 kg/m^2^ ) in contrast to those who never ate nuts (25.0 ± 0.1 kg/m^2^), despite the higher energy intakes of nuts (2191 ± 20 kcal/day compared to 1997 ± 9 kcal/day, respectively). In comparison with the carbohydrate low calorie diet, the group with almond-low calorie diet appeared 62% more greater decrease in weight, 50% in waist circumference, and 56% in fat mass and showed greater improvements in glycemic control. After eating nuts, it seems that appetite suppression occurs which may be attributed their high energy content, proteins, and fiber, which can cause a feeling of satiety. Possible causes for weight control after nuts consumption are increased satiety, energy consumption, or energy malabsorption [73].

Table 5 presents several human intervention studies about the possible effects of nuts on body weight control, obesity, and its metabolic consequences. The studies that reviewed concluded, in the most of cases, that nuts are functional foods which do not lead to increase on body weight, although they contain high fat content; however, they may lead to decreasing rhythm of weight gain or to weight loss, while they contribute to important improvements on metabolic profile, serum lipids, and glycemic control. 

### 2.6. Olive Oil

Olive oil is derived from the fruit of the olive tree (*Olea europea*) [17]. The bioactive compounds of olive oil—such as polyphenols, oleic acid, and tocopherols—have been studied as factors with possible contribution to the multifactorial metabolic and vascular protective effect afforded by Mediterranean diets. Oleuropein is the most important bioactive and phenolic compound of olive oil and its leaves [79]. The consumption of olive oil has been correlated with longevity and associated with reduction on risk of morbidity and mortality. In Mediterranean diet, olive oil has been associated with decreased risk of cardiovascular disease and cancer [80,81]. Olive oil constitutes a main source of fat, while the health benefits of olive oil consuming were attributed to the high content in oleic acid. Nowadays, it is well established that the beneficial effects of olive oil could be attributed to both oleic acid content and the phenolic fraction and has been associated with antioxidant, anti-atherosclerotic, anti-inflammatory, and anti-microbial activities [79,82].

#### Possible Effects of Olive Oil on Weight Control and Obesity’s Metabolic Consequences

The consumption of olive oil increased the body weight, as well as the waist circumference in several human studies, due its high fat content. However, the high consumption of olive oil was not correlated with higher weight gain and increased risk of overweight and obesity developing. The bioactive compounds of olive oil may contribute to a different way and rhythm of body weight gain, and olive oil consumption according the guidelines may contribute to weight management in obese patients. Several studies found that weight, waist circumference, and blood pressure decreased after olive oil consumption. Olive oil, in contrast to sunflower oil, led to decreased weight, waist circumference, blood pressure, and body fat percentages. Sunflower oil caused decline in skeletal muscle mass between the different groups. After the consumption of olive oil, the conservation of muscles and the promotion of fat tissue loss were valuable [16,83]. A randomized controlled trial observed that the consumption of 20–60 mg/day of oleuropein in powder form from 8 male rats with type II diabetes for 4 weeks led to increase in blood pressure and glucose [16]. Another randomized controlled and blinded trial concluded that the consumption of 70–398 mg of phenolics of olive oil from 20 patients with metabolic syndrome reduced cardiovascular diseases biomarkers [84]. In another randomized controlled and blinded trial the authorsobserved that the consumption of 30 mg/day polyphenols of olive oil from 24 young women with mild hypertension for 2 months decreased blood pressure [85]. Other studies showed that the consumption of 20 g/d olive oil from 93 male patients with insulin resistance led to the reduction of insulin resistance and of BMI [17,86].

A literature survey showed that although the results of the studies are contradictory, there is evidence which clearly supports the possible effect of olive oil consumption, according to dietary guidelines, on weight management, as an ideal alternative source of fat instead of saturated fats; almost all the studies show positive effect of olive oil on metabolic indices.

### 2.7. Avocado

Avocado (*Persea Americana*) is a fruit which belongs to the berry family, containing more than 400 varieties [17]. Avocado is a lipid-rich food, with provitamin A carotenoids which appeared the maximum absorption and the greater conversion to vitamin A, especially in populations with vitamin A deficiency [87]. Avocados are a bioavailable source of lutein [88] and are fruits with medium energy density, given the fact that about 80% of the avocados’ edible portion consists of water (72%) and dietary fiber (6.8%); avocado has been found to exhibit similar effects on weight management as low-fat fruits and vegetables [89]. Diets rich in avocado may improve lipoprotein profile with increase in high-density lipoprotein (HDL) and decrease in low-density lipoprotein (LDL) [88].

#### Possible Effects of Avocado on Weight Control and Health Promotion

Recent studies concluded that the consumption of 2 g of avocado pulp from 35 male rats with increased blood glucose and serum lipids for 7 weeks increased cholesterol but reduced body weight, blood glucose, BMI, and other serum lipids [17,90]. The consumption of 75 g dose or ~ ½ of a Hass avocado from 26 overweight and moderate obese adults with increased blood glucose and insulin, for 1 week at a lunch meal influenced satiety after ingestive, over subsequent periods of 3 and 5 hours. Satiety is one of the functions of the appetite system, which leads to development of completeness after a meal and decrease in hunger and restriction of further eating in the postprandial period. Specifically, a lunch meal rich in avocado led to a 23% increase in satisfaction and declined at about 28% appetite to eat over a following period of 5 hours, in contrast to meals without consumption of avocado. Furthermore, over a following period of 3 hours, the consumption of avocado led to 26% increase in satisfaction and 40% reduction on desire to eat, in contrast to a meal without avocado [91,92]. A recent study observed that 240 g dose of freeze-dried avocado pulp—when consumed by participants with type 2 diabetes mellitus, cardiovascular diseases (CVD), and cerebrovascular diseases—led to inhibition of platelet aggregation [93]. In a randomized controlled trial the authors administered 136 g doses of avocado pulp to 45 overweight and obese adults with cardiovascular disease and concluded beneficial effects on cardiovascular and metabolic risk factors [94]. In other randomized controlled trial was observed that consuming one avocado per day among a sample of 48 healthy, non-smoking women and men with macular pigment density (MPD) led to increased macular pigment density MPD levels [88].

As a conclusion from the literature survey, scientific data about the effect of avocado on body weight are limited. More studies are needed for further investigation of its consumption on weight control possibly due to their high content in fiber; however, it is clear that avocado is a fruit which can contribute to improved metabolic parameters.

Table 6 presents several human intervention studies about the possible effects of olive oil and avocado on body weight control, obesity, and its metabolic consequences.

### 2.8. Ginger

Ginger is one of the most extensively used spices and medicinal plants. Ginger is *Zingiber officinale* Roscoe and belongs to Zingiberaceae family. It is rich in volatile oils ranging 1–3%, mainly terpenoids such as zingiberene, and pungent constituents such as gingerol, shogaol, and zingerone. The main bioactive constituents of fresh and dried ginger are gingerols and shogaols [95]. Ginger may exert anti-inflammatory, anti-hypertensive, and analgesic properties and it may be used in order to reduce delayed onset muscle soreness (DOMS) after high-intensity exercise. Furthermore, several studies support that ginger could be involved in weight control, but the results are controversial [96]. In a recent study, 5 days of treatment with 4 g of ginger demonstrated a significant reduction in muscle damage biomarkers following eccentric exercise [97,98,99].

#### Possible Effects of Ginger on Weight Control: Possible Mechanisms

The exact mechanisms of the possible anti-obesity effect of ginger are not well studied. However, some possible mechanisms through which Zingiber officinal could affect body weight and body composition has been investigated [95]. Ginger may modulate obesity via various potential ways, such as increasing thermogenesis and lipolysis, suppression of lipogenesis, inhibition of intestinal fat absorption, and controlling appetite [100]. In a animal study the authors observed possible effect of 500 mg/kg of ginger for 30 days in a sample of rats with type I diabetes mellitus [18]. A randomized controlled trial concluded that 2 g of ginger when consumed from 5 men and 5 women for 5 days affected muscle damage [97]. Other study showed that the consumption of 1g ginger from 10 overweight men for 6 weeks led to increase of body weight and food consumption [96]. In a recent study was observed that consumption of 2 g of ginger by 44 patients with non alcocholic fatty liver disease for 12 weeks decreased patients’ BMI [100].

**Figure 1:**
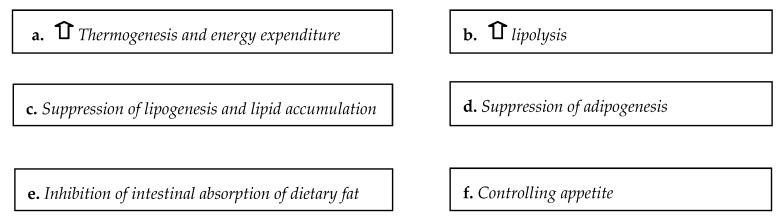
Possible mechanisms of ginger effect on body weight management.

The most studied mechanisms about the possible effect of ginger on body weight control are the following (Figure 1). (a) Increase of thermogenesis and energy expenditure: the catecholamine releasing action of ginger compounds may affect the beta adrenergic receptors and stimulate the sympathetic nerve system with the result of increased expression of uncoupling protein 1, which promotes thermogenesis [96]. (b) Increase of lipolysis: Ginger consumption may promote the activity of hormone sensitive lipase enzyme by activation of the sympathetic nerve system and thus increase the lipolysis in the white adipose tissue [101]. (c) Suppresses lipogenesis and lipid accumulation: It was reported that the bioactive compounds of ginger may exhibit antagonistic activity toward specific molecules, such as peroxisome proliferator-activated receptor sigma/beta and delta with the result of decrease of lipids accumulation in the skeletal muscle and in the adipose tissue [102]. Ginger components may also affect the expression of specific enzymes related to the lipogenesis, such as fatty acid synthase and acetyl CoA carboxylase [103]. (d) Suppresses adipogenesis: Treatment with ginger’s bioactive compounds reduced the adipogenesis in adipocytes (3T3-L1), possibly because it decreased the expression of PPARγ-associated genes. However, there are some controversial results relevant to the agonistic [103,104] or antagonistic effects of ginger on PPARγ [105,106]. (e) Inhibition of the intestinal absorption of dietary fat: It was reported in animal models that ginger may suppress pancreatic lipase enzyme; thus decreasing intestinal absorption of dietary fatty acids [95]. (f) Control of appetite: Ginger supplementation may lead to appetite attenuation [96,100]; nevertheless, the results about this mechanism are controversial [88]. Two contradictory hypotheses concerning ginger’s mechanism of action on appetite have been reported. Ginger has an inflecting effect on 5-hydroxytryptamine (5-HT; serotonin). The main elements of ginger could control the appetite, possibly through conjugation with 5-HT2c receptors in the central nervous system [97,107]. However, according to the other hypothesis, ginger may act as an appetizer through an adjusting effect on intestinal 5-HT receptors (such as 5-HT3) that raises gastrointestinal tract peristalsis and reduces the food transit time [104,108,109,110,111]; in addition, ginger consumption may reduce leptin levels [103,111,112,113].

Table 7 presents several human intervention studies about the possible effects of ginger on body weight control, obesity, and its metabolic consequences. The literature review showed that the studies are contradictory; several studies showed evidence and some mechanisms have been proposed.

## 3. Conclusions

Functional foods, when consumed as part of a balanced diet, have been proposed as a possible alternative method of weight management and obesity prevention and of improving of the metabolic consequences of obesity, including increased glucose and lipid levels. In recent years, many animal studies, as well as human clinical trials and epidemiological studies, have been performed in order to investigate the possible effect of specific functional foods and their bioactive compounds on weight control and several mechanisms of its effect have been proposed.

Several studies have shown that coffee caffeine and green tea catechins may decrease body weight, BMI, and body fat mass and help with weight control. However, the results are controversial, especially for coffee; other studies showed that the consumption of coffee increased body mass. The possible anti-obesity mechanisms of coffee and tea still need further clarification and they might have perspective as a novel strategy in order to prevent or treat obesity. In some human studies, specific kinds of berries, as well as pomegranate, led to increased fat oxidation, decreased body weight, and waist circumference, while preventing obesity’s metabolic consequences. However, more studies are needed for further investigation of berries’ anthocyanin effects on weight management. Although nuts are rich in fatty compounds, their consumption was not correlated, in the most of studies, with increased body weight, while they were associated with maintenance of body weight, helping in weight control, and increase of satiety. Furthermore, the moderate consumption of olive oil and avocado, which are also rich in fatty compounds, seems not to increase or in contrast decrease BMI, helping in weight control. Finally, studies about the consumption of ginger have shown controversial results; some studies led to increased body weight and others to reduced BMI.

To the best of our knowledge, the present review article is an attempt to critically summarize the basic findings of recent studies about the possible effect of specific functional foods and bioactive compounds on weight management and obesity’s consequences. In our opinion, this article may contribute to the better understanding of the existing research in this field and drive for further scientific investigation. However, it is not a systematic review, but a literature review article. Beyond the presented functional foods, some scientific data indicate possible effects on body control and for other foods, such as hot pepper, grapefruits, foods rich in fiber, foods rich in calcium, etc. However, in the present study, we chose to review the scientific evidence for specific functional foods that have been mentioned in a larger number of articles.

Summarizing the basic results of this review article, we could underline that, according to animal and clinical studies, specific bioactive compounds and functional foods may play a beneficial role on weight management and on attenuation of the metabolic consequences of increased body weight. However, the evidence is controversial and possible different effects have been observed from several studies. As the present literature review article indicated, most of the scientific data suggest that specific functional foods, as part of a balanced diet, could be useful in weight management and decreasing obesity’s metabolic consequences. However, their role remains unclear in most cases. Thus, more clinical and epidemiological studies are needed in order to exact safer results and conclusions and to further investigate the mechanisms of their possible effect.

## Figures and Tables

**Table 1:** Animal studies about the possible effect of functional foods and bioactive compounds on body weight control, obesity, and its metabolic consequences

Study Type (Animals Used)	Number and Characteristics of Animals	Functional Food/Dose/Duration	Summary of Key Results	Study Reference
Rats	n = 10 Male rats per 4 groups with obesity	Green tea 100 mg capsules or powder (catechins) 30 days	Reduced body weight gain	Pan et al. 2016 [15]
Rats	n = 35 male rats with high cholesterol, body weight, blood glucose, serum lipids	Avocado 2 g avocado pulp 7 weeks	Increased cholesterol Reduced body weight Reduced blood glucose Reduced BMI Reduced serum lipids	Gupta et al. 2015 [17]
Rats	n = 40 male rats with metabolic syndrome, cardiovascular, and hepatic structure	Green coffee 68.3 mg/kg caffeine 8–9 weeks	Increased cardiovascular diseases	Pauchal et al. 2012 [14]
Rats	n = 80 rats (2 groups) with chronic diseases	Berries 500 mg powder (anthocyanins) 7 weeks	Reduced chronic diseases	Nohara et al. 2018 [19]
Mice acute	n = 8–9 per group	149 g powdered berry Colitis and T cell tumournecrosis, factor-α secretion	Inhibited colitis in mice and T cell tumor necrosis factor-α secretion	Martin et al. 2018 [20]
Rats	n = 40 male rats per 5 groups with diabetes mellitus	Nuts (walnuts) 85.2 g 2 weeks	Reduced blood glucose levels	Onwuli, et al. 2014 [21]
Rats	n = 8 male rats per 5 groups with Type 2 diabetes mellitus, increased blood pressure	Olive (oleuropein) 20–60 mg/day powder RCT 4 weeks, acute	Increased blood pressure, glucose	Nekooeian et al. 2014 [16]
Dogs	n = 6 dogs with gut health	Pomegranate 50 mg powder	Positive impact on gut health	Jose et al. 2017 [22]
Rats	5 groups	Ginger 500 mg/kg, 30 days Type 1 Diabetes mellitus	Increased liver weight Decreased of plasma glucose levels	Abdulrazaq et al. 2011 [18]

**Table 2:** Human interventional clinical studies about the possible effect of coffee and caffeine on body weight control, obesity, and its metabolic consequences

Study Type/Duration	Participants/Intervention	Functional Food Dose	Summary of Key Results	Study Reference
RCT ^1^ 4 weeks	n = 142 participants	Green coffee 180 mg	Weight loss	Onakpoya et al., 2011 [31]
Acute	Women	1 cup, powder coffee Caffeine, 0.83 and 1.37 g/100 g of silverskin 300 mg powder	Prevented fat accumulation and excess weight	Martinez–Saez et al., 2014 [30]
RCT ^1^ 3 weeks	n = 25 male, 95 female with obesity	3–5 cups coffee/day	No obesity, decreased body weight, BMI, and body fat content, helping in weight control, increased number of Bifidobacterium spp.	Pan et al., 2016 [15]
RCT ^1^	n = 306 patients n = 782 adults with increased insulin (diabetes)	6 cups/day coffee	Reduced BMI Low levels of insulin	Gupta et al., 2015 [17]
RCT ^1^	n = 10 women and 12 men	caffeine 6 mg	Increased body mass	Laurence et al., 2012. [32]
RCT ^1^	n = 10 Males, 18–50 years old, with Type II diabetes mellitus	3–4 cups coffee per day acute	Glucose control improved	Moisey et al., 2009 [33]
RCBT ^2^ acute	n = 10 men	Caffeine 80 mg	Appetite control	Schubert et al., 2017 [34]
RCBT ^2^ acute	n = 10 Men with increased glucose, insulin	5 mg caffeine	Decreased glucose, insulin	Beaudoin et al., 2011 [28]
RCT ^1^ acute	15% women with osteoporosis and 51% with low bone mass 4% men with osteoporosis and 35% with low bone mass	Caffeine 400 mg Capsule or powder	No association with increased risk of chronic diseases in healthy adults (premature death, cardiovascular diseases, and cancer)	US Dietary Guidelines Advisory Committee (DGAC), 2015 [27]
RCBT ^2^ acute 2 months RCT ^1^ acute	n = 137 Patients with Arrhythmic episodes	Caffeine 500 mg capsules or powder Caffeine 35 mg capsules or powder Decaffeinated coffee 100 mL or 4 cups/day capsules or powder	No arrhythmic episodes	Zuchinali et al., 2016 [29]
RCT ^1^	n = 9 healthy participants	1–5 cups/day coffee	Limited plasma appearance of bioactives and metabolites of coffee	Renouf et al., 2010 [35]

^1^ RCT: randomized control trials, ^2^ RCBT: randomized control blinded trials.

**Table 3:** Human intervention clinical studies about the possible effects of green tea on body weight control, obesity and its metabolic consequences

Study Type/Duration	Participants/Intervention	Functional Food Dose	Summary of Key Results	Study Reference
RCBT ^2^ 12 weeks acute	n = 115 obese women	Catechins 491 mg capsules	Weight reduction	Chen et al., 2016 [48]
RCT ^1^ acute	n = 8821 adults with obesity and increased diastolic blood pressure n = 35 obese people	3 cups/day capsules green tea 4 cups/day capsules green tea	Lower BMI Higher diastolic blood pressure BMI decreased	Basu, et al., 2010 [49]
RCT ^1^ acute	n = 24 participants women 23–32 years old	4–5 cups capsules green tea	BMI normal levels	Egert et al., 2012 [50]
RCT ^1^ acute	n = 159 human (adults) with hypatotoxicity risk, thyroid toxicity	3 cups/day capsules green tea catechins 304 mg	Subchronic-toxicity carcinogenicity thyroid toxicity	Hu et al., 2018 [36]
RCBT ^2^ 14 days 14 weeks acute	n = 40 male n = 37 female with liver problems fasting plasma glucose, hepatotoxicity	Catechins 704 mg 1 cup green tea	No adverse effects on liver no affect fasting plasma glucose No hepatotoxic effect	Toolsee et al., 2013 [47]
RCT ^1^	n = 8 young men	Catechins (EGCG) epigallocachin-3 gallate 90 mg capsules caffeine 50 mg capsules	Stimulate thermogenesis	Gosselin et al., 2012 [43]
RCBT ^2^	n = 18 patients (men) with muscle metabolism	Catechins epigallocachin-3 gallate (EGCG) 600 mg capsules	Improved muscle metabolism	Mähler, et al., 2015 [51]
RCT ^1^ acute 6 months	n = 43 patients with coronary artery disease	4.5 g green tea	Did not affect coronary artery disease decreased postprandial triglycerides increase	Koutelidakis et al., 2013 [52]
RCT ^1^ acute	n = 5 female, n = 4 male with obesity, metabolic syndrome	4 cups/day capsules green tea	Not significantly affected features of metabolic syndrome	Basu et al., 2011 [53]
RCBT ^2^ 7 days	90 obese people 30 overweight people	Retroperitoneal, epididymal, mesenteric adipose tissues	Reduced retroperitoneal, epididymal, mesenteric adipose tissues	Pan et al., 2016 [15]
RCBT ^2^ 7 days	90 obese people 30 overweigh people	Catechins 68.99 mg capsules or powder	Reduced retroperitoneal, epididymal, mesenteric adipose tissues	Yamashita et al., 2014 [54]

^1^ RCT: randomized control trials. ^2^ RCBT: randomized control blinded trials.

**Table 4:** Human intervention clinical studies about the possible effects of berries and pomegranate on body weight control, obesity, and its metabolic consequences

Study Type/Duration	Participants/Intervention	Functional Food Dose	Summary of Key Results	Study Reference
RCT ^1^ One week	n = 27 overweight or obese men	Berries (Blackberry) 600 g	Increased fat oxidation reduced insulin sensitivity increased hepatic glucose	Solverson et al., 2018 [61]
RCT ^1^ 35 days acute	n = 101 overweight or obese women with metabolic syndrome	100 g powdered berries	Decreased waist circumference and body weight—positive effects on metabolic diseases	Lehtonen et al., 2011 [68]
RCT ^1^ 20 weeks acute	n = 61 women with decreased fasting plasma HDL-C and systolic and diastolic blood pressure	163 g Berries	Increased fasting plasma HDL-C decreased systolic and diastolic blood pressures	Lehtonen et al., 2010 [59]
RCBT ^2^ 12 weeks acute	n = 63 participants adults 20–79 years with diabetes mellitus, decreased glycemic control, increased fasting glucose	Berries 100–140 mg/dL in capsules	Increased glycemic control, decreased fasting glucose	Choi et al., 2017 [58]
RCT ^1^ 5 weeks	n = 40 healthy older adults 50–70 years old with increased cardio-metabolic risk markers	Anthocyanins 414.2 mg/L from berries	Improvements in type II diabetes mellitus and cardiovascular disease biomarkers	Nilsson et al., 2017 [57]
RCT ^1^ acute	n = 21.13 million people metabolic syndrome	Pomegranate leaf extract and ascorbic acid 10–20 mg in capsules or powder	Treatment of obesity and type 2 diabetes mellitus reduced body weight	Medjakovic et al., 2013 [67]

^1^ RCT: randomized control trials; ^2^ RCBT: randomized control blinded trials.

**Table 5:** Human intervention clinical studies about the possible effects of nuts on body weight control, obesity and its metabolic consequences

Study Type/Duration	Participants/Intervention	Functional Food Dose	Summary of Key Results	Study Reference
RCT ^1^ 4 weeks	n = 46 (28 women, 18 men) overweight, obese adults	Walnuts 56 g	Improved endothelial function in overweight	Katz MD et al., 2012 [77]
RCT ^1^ acute	n = 21 Men 45–75 years with prostate cancer and overweight	Walnuts 75 g/day	Maintained body weight	Kranz et al., 2013 [74]
RCT ^1^ 12 weeks	n = 300 adults 51 years with type 2 diabetes mellitus	Cashew nuts 30 g/day	Increased body weight, BMI, waist circumference, increased HDL cholesterol and reduced systolic blood pressure	Mohan et al., 2018 [76]
RCT ^1^	n = 8800 (men, women) obese, type 2 diabetes mellitus	67 g nuts	Reduced insulin levels, reduced LDL cholesterol, and increased HDL cholesterol	Ros et al., 2010 [69]
RCT ^1^ acute	n = 63 patients with prostate cancer or prostate hyperplasia	Walnuts 50 g	Improved prostate biomarkers	Sánchez-González et al., 2015 [72]
RCT ^1^ 12 week sacute	n = 50 patients with insulin resistance	30 g nuts	Decreased insulin resistance	Casas-Agustench et al., 2011 [75]
RCT ^1^ 4 weeks acute	n = 137 participants with postprandial glycemia	Almonds 43 g	Suppressed hunger	Tan and Matteset al., 2013 [78]

^1^ RCT: randomized control trials.

**Table 6:** Human intervention clinical studies about the possible effects of olive oil and avocado on weight control, obesity, and its metabolic consequences.

Study Type/Duration	Participants/Intervention	Functional Food Dose	Summary of Key Results	Study Reference
RCT ^1^ 6 months	n = 93 male patients with insulin resistance	Olive oil 20 g/day	Reduction in BMI and increased insulin sensitivity	Gupta et al., 2015 [17] Nigam P et al., 2014 [86]
RCBT ^2^ 1 week	n = 26 healthy overweight adults with increased blood glucose and insulin	Avocado 75 g or ½ avocado	Insulin resistant varied BMI	Wien et al., 2013 [92]
RCBT ^2^ acute	n = 20 patients with metabolic syndrome	Phenolics (olive oil) 70–398 mg	Reduced cardiovascular disease	Camargo et al., 2010 [84]
RCBT ^2^ 2 months	n = 24 young women with mild hypertension	Polyphenols (olive oil) 30 mg/day	Decrease blood pressure	Moreno-Luna et al., 2012 [85]
2 weeks	3 Male adult mice per group type 2 diabetes cardiovascular diseases (CVD) platelet aggregation	240 g freeze-dried avocado pulp	Inhibited platelet aggregation	Rodriguez-Sanchez et al., 2015 [93]
RCT ^1^	n = 45 overweight and obese adults with Cardiovascular disease	136 g avocado pulp	Beneficial effects on cardiovascular and metabolic risk factors	Wang et al., 2015 [94]
RCT ^1^ 6 months	n = 48 healthy, non-smoking women and men with macular pigment density (MPD)	Avocado	Increased MPD levels	Scott et al., 2017 [88]

^1^ RCT: randomized control trials; ^2^ RCBT: randomized control blinded trials.

**Table 7:** Human intervention clinical studies about the possible effects of ginger on body weight control, obesity, and its metabolic consequences

Study Type/Duration	Participants/Intervention	Functional Food Dose	Summary of Key Results	Study Reference
RCT ^1^ 6 weeks acute	n = 10 overweight men	1 g ginger	Increased body weight and food consumption	Mansour et al., 2012 [96]
RCT ^1^ 12 weeks	n = 44 patients with non-alcoholic fatty liver disease	2 g ginger	Decreased BMI	Attari et al., 2017 [100]
RCT ^1^ 5 days	n = 5 men and n = 5 women with muscle damage	2 g ginger	Increased muscle damage	Matsumura et al., 2015 [97]

^1^ RCT: randomized control trials.
